# Serial dependence: A matter of memory load

**DOI:** 10.1016/j.heliyon.2024.e33977

**Published:** 2024-07-02

**Authors:** Yuri A. Markov, Natalia A. Tiurina, David Pascucci

**Affiliations:** a Laboratory of Psychophysics, Brain Mind Institute, École Polytechnique Fédérale de Lausanne (EPFL), Switzerland; b Department of Psychology, Goethe University Frankfurt, Frankfurt Am Main, Germany; c Department of Psychology, TUD Dresden University of Technology, Dresden, Germany; d The Radiology Department, Lausanne University Hospital and University of Lausanne, Lausanne, Switzerland; e The Sense Innovation and Research Center, Lausanne and Sion, Lausanne, Switzerland

**Keywords:** Serial dependence, Visual working memory, Memory load, Perceptual history, Serial biases

## Abstract

In serial dependence, perceptual decisions are biased towards stimuli encountered in the recent past. Here, we investigate whether and how serial dependence is affected by the availability of visual working memory (VWM) resources. In two experiments, participants reproduced the orientation of a series of stimuli. On alternating trials, we included an additional VWM task with randomly varying levels of load. Serial dependence was not only affected by the additional load task but also clearly modulated by the level of load: a high load in the previous trial reduced serial dependence while a high load in the present increased it. These results were independent of the effects of VWM load on the precision of reproduction responses. Our findings provide new insights into the mechanisms that may regulate serial dependence, revealing its intimate link with VWM resources.

**Significance statement:**

Our perception, thoughts, and behavior are continuously influenced by recent events. For instance, the way we process and understand current visual information depends on what we have seen in the preceding seconds, a phenomenon known as serial dependence. The precise mechanisms and factors involved in serial dependence are still unclear. Here, we demonstrated that working memory resources are a crucial component. Specifically, when we are currently experiencing a heavy memory load, the influence of prior stimuli becomes stronger. Conversely, when prior stimuli were shown under a high memory load, their influence was reduced. These findings highlight the importance of working memory resources in shaping our interpretation of the present based on the recent past.

## 1 Introduction

Prior history can influence how we perceive, make decisions, and behave, as evident from findings in nearly all domains of psychology and psychophysics research [[1], [2], [3], [4], [5], [6], [7], [8], [9]]. In recent years, research on *serial dependence* has provided some of the most compelling examples of these phenomena, revealing that perceptual decisions are systematically attracted towards features of stimuli seen before ([10]; see Ref. [11,12] for reviews).

One major focus has been on investigating serial dependence in perceptual tasks involving basic visual features, such as stimulus orientation [10,[13], [14], [15], [16], [17], [18], [19], [20], [21], [22], [23], [24], [25], [26], [27], [28], [29], [30], [31], [32]]. In these tasks, participants are asked to reproduce the orientation of stimuli presented in a series of trials, and their responses are biased towards the orientation of the stimulus shown in the preceding trial.

While serial dependence effects have been reported across nearly all sorts of perceptual tasks, suggesting a general trait of human perception and cognition [14,28,[33], [34], [35], [36], [37], [38], [39], [40]], the underlying mechanisms remain a subject of debate. One central question revolves around the level of processing involved in serial dependence and the extent to which post-perceptual and task-related factors, such as decision-making and memory, contribute. This debate traces back to early literature on sequential effects in psychology and psychophysics (see Ref. [12] for review), and has led to the emergence of two main perspectives, often referred to as the perceptual and post-perceptual accounts.

The perceptual account suggests that attractive serial dependence—the bias *towards* prior stimuli—results from mechanisms that operate at the perceptual level, where similar stimuli occurring closely in space and time are automatically integrated to maintain perceptual continuity and stability [10,11,19,36,41,42]. According to this view, these mechanisms should function independently of higher-level cognitive functions like memory and decision-making [3,10,14,19,35,36,[41], [42], [43], [44]]. This perspective is supported by studies demonstrating that attractive serial dependence persists even in the absence of an explicit task on the previous trial, and despite participants cannot recall features of previous stimuli from working memory [10,19,27,35,40,[44], [45], [46], [47]].

In contrast, the post-perceptual account argues that attractive serial dependence originates at higher-level cognitive stages [16,17,20,23,28,29,34,48,49], emphasizing the role of visual working memory (VWM) [20,[50], [51], [52], [53], [54]]. Research in this framework shows that the strength of serial dependence is modulated during working memory maintenance, and memory circuits in the prefrontal cortex may play a direct causal role [20,52,55,55,56]. Thus, the post-perceptual account suggests that attractive serial dependence is influenced by aspects related to task execution, such as decision-making templates and working memory representations [17,20,23,28,34,52], whereas recent stimulus history tends to have *repulsive* effects, causing biases in perception away from previous stimuli [16,17,20,21,28,29,34,49,51,[57], [58], [59], [60], [61], [62], [63]].

The debate between perceptual and post-perceptual accounts extends to neuroimaging research as well. While initial investigations supported the emergence of attractive serial dependence in the primary visual cortex (V1) [64], recent work suggests that neural representations in V1 are primarily repelled away from previous stimuli, whereas attractive serial dependence originates at higher-level processing stages [61]. Consistent with this view, direct attempts to investigate the sources of serial dependence, such as via Transcranial Magnetic Stimulation (TMS), point to a causal role of memory circuits in the prefrontal and posterior parietal cortex [50,56,65].

These contrasting perspectives have not only shaped the framing and interpretation of individual studies but also influenced computational and theoretical models of serial dependence [10,50,53]. Research has predominantly focused on either perception or VWM, with attempts to reconcile the two suggesting that serial dependence may operate at multiple interacting levels [3,66]. Yet, mixed results prevent a clear understanding of the nature of serial dependence and the involvement of VWM.

The literature reviewed above suggests that maintaining the current stimulus in VWM for an extended period can amplify the bias towards prior stimuli [20,52]. Conversely, disrupting VWM traces of previous stimuli can weaken the strength of serial dependence [56]. This indicates that the quality of both current and prior VWM representations plays at least an important role in serial dependence.

A straightforward way to test this hypothesis is by manipulating VWM load during a sequence of perceptual decisions. Recent studies have shown that increasing VWM load can detrimentally affect both the maintenance and fidelity of memory representations [67], impacting well-known phenomena that depend on recent history, such as contextual cueing, priming, and predictive effects [4,5,7,[68], [69], [70], [71], [72], [73], [74], [75], [76], [77], [78]]. Thus, it is possible that even in classic perceptual tasks with basic features like orientation, increasing VWM load may differentially affect the persistence of prior stimulus information and the current tendency to be biased by recent stimulus history. As of yet, these predictions, key for gaining deeper insights into the interplay between VWM and serial dependence, have not been exhaustively tested.

In this study, we investigated the effects of loading VWM during a classic paradigm of serial dependence in orientation perception. Participants reproduced the orientation of a central Gabor patch in a series of trials. On alternating trials, we introduced a concomitant VWM task with randomly varying levels of load. By manipulating VWM load on either the previous or current trial, we aimed to assess whether and how VWM load impacts serial dependence, by selectively affecting the memory trace of the previous or of the current stimulus.

Overall, we found that serial dependence increased when the present trial involved an additional memory task. Importantly, the level of load had a clear modulatory effect: a high memory load in the previous trial reduced serial dependence, while a high memory load in the present increased it. These effects were independent of the influence of VWM load on performance and reproduction precision.

Our findings provide novel evidence of a clear interdependence between VWM and serial dependence, with implications for current models and future research directions.

## 2 Results

### 2.1 Experiment 1

**Fig. 1:**
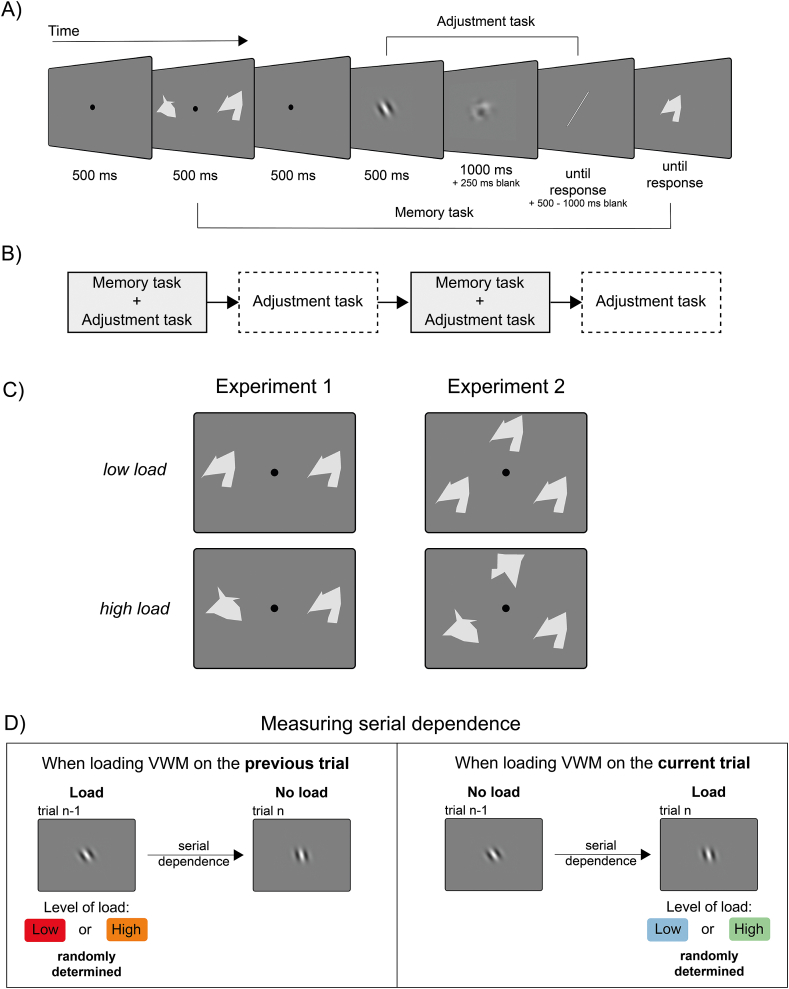
Experimental design. A) Sequence of events in one trial comprising both the memory and the adjustment task (*load* condition). Observers performed the orientation task while holding in memory two abstract shapes. At the end of the trial, they had to report whether a test shape was present or not in the memory display. B) Trials comprising both the memory and the adjustment task were alternated with trials with only the adjustment task (*no-load* condition, see Methods). C) The level of load in *load* trials was manipulated by presenting identical shapes (*low load*, 2 shapes in Experiment 1, 3 in Experiment 2) or all different shapes (*high load*). D) Measuring serial dependence as a function of the level of VWM load on the previous (left panel) or current (right panel) trial. Note that, while *load* and *no-load* trials were regularly alternating, the level of load was randomly determined. The two conditions highlighted in D) also imply that when load was applied to the previous trial, the current trials contained only a single task: the orientation reproduction task; conversely, when load was applied to the current trial, the current trial contained a dual task, with the memory task about shapes plus the orientation reproduction task. The color coding of the *low* and *high load* differs depending on whether the *load* was applied on the previous (red and orange) or current (blue and green) trial, and this color scheme is consistent with the results figure. Stimuli are not drawn to scale. (For interpretation of the references to color in this figure legend, the reader is referred to the Web version of this article.)

Participants reproduced the perceived orientation of a series of Gabor patches (Fig. 1). On alternating trials, an additional memory task was introduced, requiring participants to memorize two peripheral abstract shapes (*load* condition). The level of VWM load varied, with either two identical (*low load*) or two different shapes (*high load*), randomly determined (e.g., participants could not anticipate the load demand).

During trials with the memory task, the presentation of the Gabor patch followed the presentation of the two memory items (Fig. 1A). After adjusting the orientation of the response bar, participants were presented with a single test shape (old vs. new task).

During trials without the memory task (*no-load* condition), participants simply reproduced the orientation of the central Gabor patch, as in traditional serial dependence paradigms. This design was tailored specifically to investigate 1) serial dependence in relation to the level of load (*high* vs. *low*) on the previous trial and 2) in relation to the level of load on the current trial (Fig. 1C).

**Fig. 2:**
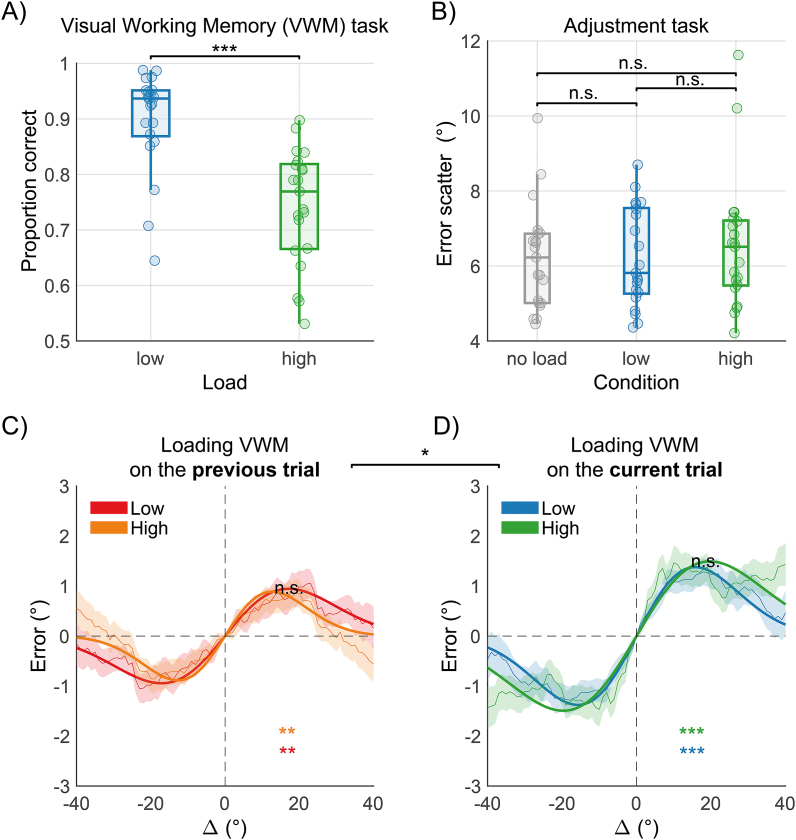
Result of Experiment 1. A) Boxplots with individual data points illustrating the performance in the *low* (blue) and *high load* (green) memory task conditions. B) Boxplots showing the error scatter in the adjustment task (the standard deviation of adjustment errors) as a function of the load condition. *No load* refers to trials where the adjustment task was completed without any VWM demand (grey), while *low* and *high* indicate the level of load in *load* trials, where an additional VWM task was presented (in blue and green, respectively). C) Serial dependence effects when loading VWM on the previous trial (single task on the current trial). The red and orange colors represent conditions where the previous trial contained *low* or *high* VWM load, respectively (see also Fig. 1D). D) Serial dependence effects when loading VWM on the current trial (dual task on the current trial). Blue and green colors indicate *low* or *high* VWM load, respectively (see also Fig. 1D). In panels C and D, Δ refers to the acute angle difference between the previous and current stimulus orientation. Curves are circular running averages with 1 standard deviation of the aggregate data of all subjects. Fits are derived from the best-fitting δoG (see Methods). Asterisks denote the significance of the half-amplitude parameter against 0 with the respective color coding of each condition. "N.s." indicates a non-significant difference between conditions, whereas the asterisks connecting C and D indicate a significant difference overall between the half-amplitude of serial dependence when loading VWM on the previous (*no load* on the current) or on the current (*load*) trial. * = *p* < 0.05, ** = *p* < 0.01, *** = *p* < 0.001. (For interpretation of the references to color in this figure legend, the reader is referred to the Web version of this article.)

To examine performance in the VWM task, we compared the proportion of correct responses (pc) under *low* and *high load* trials. Participants performed well above chance in both the *low load* (pc = 0.899 ± 0.091) and *high load* (pc = 0.744 ± 0.104) conditions, with significantly better performance in the *low load* condition (t(20) = 10.11, *p* < 0.001, Cohen's *d’* = 2.20, BF_10_ > 100, Fig. 2A).

We then evaluated the impact of the VWM task on adjustment performance by comparing the standard deviation (error scatter) of adjustment errors, cleaned from systematic bias and serial dependence effects (see Methods), between *load* and *no-load* trials and between the *low* and *high load* conditions in *load* trials (Fig. 2B). The presence of a memory task did not affect performance in the adjustment task, with comparable performance in trials that required memorization of shapes (error scatter = 6.403 ± 1.387°) compared to trials without a memory demand (error scatter = 6.212 ± 1.391°, t(20) = −0.94, *p* = 0.356, *d’* = −0.20, BF_10_ = 0.34). Similarly, in *load* trials, performance in the adjustment task showed no difference between *low* and *high* VWM load conditions (*low load* error scatter = 6.242 ± 1.312, *high load* error scatter = 6.559 ± 1.744, t(20) = −0.99, *p* = 0.334, *d’* = −0.22, BF_10_ = 0.35).

In the analysis of serial dependence, we focused on how the orientation of the previous stimulus affected current adjustment errors, considering the presence and level of load on the current or preceding trials (Fig. 1D). Serial dependence in orientation typically results in a bias towards previous stimuli when the orientation difference (Δ) between previous and current stimuli is small (e.g., less than 45°). This bias was quantified using a 1st derivative of a Gaussian function (δoG, see Methods), with the parameters indicating the magnitude (half-amplitude) and extent (width) of the bias across different Δ values. Specifically, the half-amplitude quantifies the peak bias, measured in degrees, of current errors towards the orientation presented in the preceding adjustment trials; whereas the width parameter indicates the spread of the bias across the range of possible orientation differences, with smaller widths suggesting a bias limited to highly similar past and present orientations.

When assessing the effects of VWM load on the previous trial, we observed significant serial dependence in both *low* (half-amplitude = 0.95°, *p*_*perm*_ = 0.007; see Methods for the permutation approach) and *high* load (half-amplitude = 0.89°, *p*_*perm*_ = 0.009), with no significant difference between the two (half-amplitude difference (*low* minus *high*) = 0.06°, *p*_*perm*_ = 0.443, width difference = −0.01, *p*_*perm*_ = 0.134; Fig. 2C).

Similarly, when examining the effects of VWM load on the current trial, we found clear serial dependence in both *low* (half-amplitude = 1.38°, *p*_*perm*_ < 0.001) and *high* load conditions (half-amplitude = 1.49°, *p*_*perm*_ < 0.001), with no significant difference (difference in half-amplitude = −0.12°, *p*_*perm*_ = 0.391; difference in width = 0.01, *p*_*perm*_ = 0.281; Fig. 2D). Serial dependence was overall larger in trials involving a VWM load compared to trials without load (*no-load* half-amplitude = 0.92°, *p*_*perm*_ < 0.001; *load* half-amplitude = 1.44°, *p*_*perm*_ < 0.001; difference in half-amplitude = −0.53°, *p*_*perm*_ = 0.034, difference in width = 0.01, *p*_*perm*_ = 0.221). These results were further validated using a non-parametric approach (see Supplementary Material).

### 2.2 Experiment 2

The results of Experiment 1 indicated that the level of load (*low* or *high*), whether applied to the previous or current trial, did not produce any significant effect. However, the presence of a load task did impact the magnitude of serial dependence, despite no observable influence on overall performance levels (error scatter) in the adjustment task. One potential explanation is that while loading VWM may indeed have a significant effect on serial dependence, the level manipulation used—memorizing two identical versus two different items—may not have been sufficiently robust to detect a clear effect of load level. Given that the presence of the load task could be anticipated, its precise effect remains unclear. Therefore, in Experiment 2, we increased the level of load in the *high* load condition by presenting three different shapes (Fig. 1C).

**Fig. 3:**
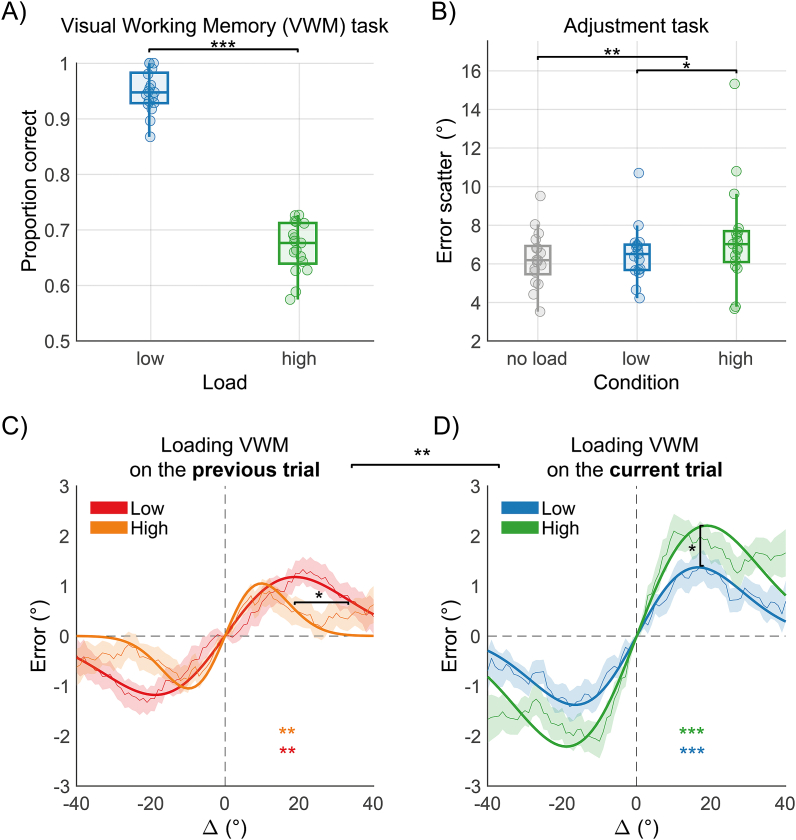
Result of Experiment 3. A) Boxplots with individual data points illustrating the performance in the *low* (blue) and *high load* (green) memory task conditions. B) Boxplots showing the error scatter in the adjustment task (the standard deviation of adjustment errors) as a function of the load condition. C) Serial dependence effects observed when loading VWM on the previous trial (single task on the current trial). D) Serial dependence effects observed when loading VWM on the current trial (dual task on the current trial). Color coding is the same as in Fig. 2. The asterisk in C) denotes a significant difference in width (see Results). * = *p* < 0.05, ** = *p* < 0.01, *** = *p* < 0.001. (For interpretation of the references to color in this figure legend, the reader is referred to the Web version of this article.)

As in Experiment 1, participants performed the memory task in the *low load* (pc = 0.949 ± 0.037) and *high load* conditions (pc = 0.668 ± 0.046) with significantly better performance in the *low load* condition (t(16) = 24.33, *p* < 0.001, *d’* = 5.9, BF_10_ > 100, Fig. 3A). However, adjustment performance in this experiment deteriorated in the presence of *load* (error scatter = 6.962 ± 2.039°) compared to *no-load* trials (error scatter = 6.253 ± 1.417°; t(16) = −3.18, *p* = 0.005, *d’* = −0.77, BF_10_ = 8.42), in contrast to the absence of differences observed in Experiment 1. Nonetheless, only anecdotal evidence supported a difference between the *low* and *high* VWM load conditions (*low load* error scatter = 6.532 ± 1.430, *high load* error scatter = 7.386 ± 2.681, t(16) = −2.28, *p* = 0.036, *d’* = −0.55, BF_10_ = 1.88, Fig. 3B, see Methods for the interpretation of 10.13039/501100021785BF).

When VWM load was applied on the previous trial, serial dependence was observed in both *low* (half-amplitude = 1.18°, *p*_*perm*_ = 0.001) and *high* load trials (half-amplitude = 1.05°, *p*_*perm*_ = 0.009), with no significant difference in the half-amplitude (*low* minus *high* = 0.13°, *p*_*perm*_ = 0.356). However, we found a significant difference in width (difference = −0.03, *p*_*perm*_ = 0.016; Fig. 3C), indicating a broader range of the effect of previous stimuli when the previous trial involved *low load*.

Serial dependence was also evident when examining the effects of VWM load on the current trial, in both *low* (half-amplitude = 1.38°, *p*_*perm*_ < 0.001) and *high* load conditions (half-amplitude = 2.21°, *p*_*perm*_ < 0.001). Importantly, the two conditions were significantly different in the estimated half-amplitude (difference in half-amplitude = −0.83°, *p*_*perm*_ = 0.029; difference in width = 0.01, *p*_*perm*_ = 0.339; Fig. 3D). As observed in Experiment 1, serial dependence was overall larger in trials involving VWM load compared to trials without load (*no-load* half-amplitude = 0.96°, *p*_*perm*_ < 0.001; *load* half-amplitude = 1.81°, *p*_*perm*_ < 0.001; difference in half-amplitude = −0.84°, *p*_*perm*_ = 0.001, difference in width = 0.001, *p*_*perm*_ = 0.478; see Supplementary Material for non-parametric validation).

Therefore, compared to Experiment 1, increasing the load demand in the *high load* condition of Experiment 2 led to clear modulations of serial dependence. Importantly, additional control analyses demonstrated that these effects were not driven by participants allocating attentional resources differently between load levels (see Supplementary Material and Supplementary Fig. 1).

## 3 Discussion

We investigated the impact of VWM load on serial dependence by manipulating the load level (*low* or *high*) in either the previous or current trial. Across two experiments, we consistently observed an increase in the strength of serial dependence when participants engaged in a VWM task during the current trial. Moreover, in Experiment 2, where the *high load* condition imposed greater VWM demand, we observed a clear modulatory effect of load level: Serial dependence decreased when previous stimuli were presented under high load, whereas it increased when current stimuli were presented under high load.

### 3.1 VWM load modulates serial dependence

Our findings contribute to and expand upon previous research demonstrating the modulatory effects of VWM retention intervals on serial dependence [20,52], suggesting a more fundamental role of VWM resources.

One possibility is that both the effects of load and retention intervals stem from similar VWM dynamics that manifest when the current memory trace is subject to deterioration. For instance, according to models of attractor dynamics in VWM, as the memory of the current stimulus deteriorates over longer retention intervals or under increased load demand, it tends to shift towards stable neural states — ‘local attractors’ [67]. These local attractors likely reflect memory representations of recently encountered stimuli [28,50,67]. Thus, in our experiments, particularly in the high load condition of Experiment 2, the drift towards prior memory traces may have been accelerated by the higher load demand, resulting in stronger serial dependence. This aligns with the predictions of recent drift-diffusion models of VWM that consider the impact of load [67].

In a similar vein, stronger deterioration of memory traces under load could explain the reduced influence of prior stimuli when load was imposed on the preceding trial (see Fig. 3C). Namely, load-induced deterioration may have more rapidly erased the previous stimulus trace once active maintenance was no longer necessary after the response. This likely accounts for why performance remained relatively consistent across load conditions, despite variations in serial dependence.

The effects of loading VWM on the current trial also fit well with models that emphasize the role of noise and propose that memory representations embed full likelihood distributions across neuron populations [79]. Under specific memory states, such as high load conditions, a broader likelihood distribution may more frequently lead to the interference of prior memory traces with current ones [[80], [81], [82], [83]].

Although the exact neural mechanisms should be better clarified by future studies, our data suggest that VWM resources might have a determinant or even causal role in the strength of serial dependence, challenging the idea of complete independence between the two.

Another aspect worth mentioning in favor of the role of VWM is that, due to the structure of the task, in trials involving only the reproduction response (no-load), the last event on the preceding trials involved recalling the shape(s) in VWM for comparison with the test shape. Conversely, in trials involving the additional VWM task (load), the last event on the preceding trial was the reactivation of the orientation kept in memory for reproduction (Fig. 1). Thus, in this latter case, the memory of the previous orientation, rather than the perception of it, was more recent and stronger than in the former case, potentially explaining the stronger serial dependence in load compared to no-load trials. We propose that future research may test variations of this paradigm and the event structure to further evaluate the contribution of the last event on the preceding trial in modulating serial dependence.

### 3.2 VWM load affects serial dependence without altering error variability

Our study consistently revealed an increase in serial dependence in trials involving a concurrent VWM task. At first, one might interpret this as the mere effect of dual-task demand, arguing that the additional task diverts resources away from the Gabor stimulus, thereby potentially degrading its perception and memory [[84], [85], [86], [87]]. This increased uncertainty could theoretically lead to larger serial dependence [12,16,32,88].

However, if this were the case, one would expect a similar effect on the precision of adjustment reports, or 'error scatter', which typically increases with uncertainty [19]. Contrary to this, our experiments did not reveal such a pattern. In both experiments, we either found no evidence of an effect of load (and load level) on the error scatter (Experiment 1) or only inconclusive results (Experiment 2). Furthermore, the manipulation of load level in Experiment 2 produced unequivocal effects on serial dependence, which persisted even among participants exhibiting completely comparable performance in the two conditions (see Supplementary Material). Hence, our findings are unlikely due to the differential processing of Gabor stimuli under dual-task conditions.

It is worth noting that our study is not the first to demonstrate that targeted manipulations can modulate serial dependence without affecting accuracy and precision in performance. Similar findings have been reported in investigations of VWM with more direct manipulations. In the study of de Azevedo Neto and colleagues, for instance, TMS applied to memory-related regions disrupted serial dependence without deteriorating performance [56]. Taken together, these findings also challenge an unequivocal and mandatory relationship between bias and precision in serial dependence [19].

### 3.3 The relationship between VWM and serial dependence

Despite the recent surge in studies on serial dependence, there are still clearly diverging research trajectories (see Ref. [11,12,50,76,89] for different perspectives and reviews). As noted earlier, some studies emphasize the perceptual component of serial dependence and advocate for specialized mechanisms in perception [19,21,26,41,90], while others contextualize these phenomena within the framework of working memory biases [34,50,57,91,92] and the dynamics of neural attractors in prefrontal circuits [50,53,93].

Within this debate, it is important to note that the effects reported here seem to have little to do with early perceptual mechanisms. For example, VWM was loaded with abstract shapes different from orientations, and the memory items were presented at different locations from the adjustment stimuli. Thus, our findings cannot be directly attributed to interactions at low-level processing stages, such as orientation-selective retinotopic responses in the primary visual cortex [16]. Instead, our results suggest the involvement of more general cognitive states, including a high load demand, in modulating the strength of serial dependence irrespective of the stimuli involved [88]. In the context of our research question, we propose that to counteract the detrimental effects of high load on the concurrent task, observers may exhibit a stronger tendency towards an 'ensemble' type of information processing [[94], [95], [96], [97], [98], [99]]. This involves integrating the memory representation of the current stimulus with past information to maintain more precise, albeit biased, representations. Such a process can be framed within Bayesian and optimal integration strategies, wherein load affects the reliability and relative weight of prior and current VWM representations in perceptual decisions ([19,21]; but see Ref. [16,22]).

While the exact computational principles behind serial dependence are beyond the scope of this study, it is possible that in our paradigm, modulations of serial dependence served to counteract the noise introduced by load manipulation. This could also explain why effects prominently observed in the strength of serial dependence were not consistently reflected in the precision of adjustment responses. This observation aligns with previous findings demonstrating that aspects beyond the stimulus, such as the level of confidence in the report made can modulate serial dependence independently of overall task performance ([30]; see also [88]).

One might question how these findings relate to other, more nuanced, perspectives on serial dependence, which propose that decision-related processes play a primary role [15,16,23,28,29,49]. VWM naturally takes the forefront in preparing for decision-making or action execution [100]. Hence, VWM and decision-making may be complementary processes in the causal chain leading to serial dependence: the demand for a decision engages VWM, but the quality and availability of VWM resources determine the persistence and strength of prior memories.

While compartmental views of perception, memory, and decision-making are reductive, serial dependence may well involve interactions across multiple processing stages [3], targeted manipulations, such as the one presented in our study, are crucial for understanding the functioning of these phenomena. For instance, proponents of a perceptual mechanism often emphasize the broad temporal, spatial, and featural tuning of these effects (see Ref. [11] for a review and meta-analysis). However, these characteristics are not exclusive to low-level perceptual mechanisms, as they are also evident when considering higher-level processing stages [50,53,97,[101], [102], [103], [104], [105], [106], [107], [108]]. Conversely, susceptibility to VWM load emerges as a defining feature, suggesting that serial dependence is contingent upon specific states, including different levels of VWM load, rather than being governed by a fixed mechanism (see also [88]).

## 4 Conclusion

Serial dependence is ubiquitous in nearly every perceptual and cognitive task. Yet, the extent to which these phenomena are influenced or can be modulated by working memory processes remains debated. Here, we present compelling evidence of the impact of VWM load on serial dependence, supporting a causal relationship between VWM resources and serial dependence [56]. We propose that under high VWM load, memory traces, which experience more pronounced deterioration, exhibit a greater tendency to drift towards prior memory representations. These findings offer new insights and fertile ground for the development of new and refined models of serial dependence.

## 5 Methods

### 5.1 Ethics statement

The study was approved by the local ethics committee under the Declaration of Helsinki (except for preregistration) (World Medical Organization, 2013). This study was not preregistered.

### 5.2 Apparatus

Stimuli were presented on a gamma-corrected VG248QE monitor (resolution: 1920 x 1080 pixels, refresh rate: 120 Hz) and were generated with custom-made scripts written in Matlab (R2013a) and the Psychophysics Toolbox [109], on a Windows-based machine. Participants sat at approximately 57 cm from the computer screen, with the head held stable in a chin rest. The experiments were performed in a dim room.

### 5.3 Participants

In total 38 healthy human participants from the EPFL and the University of Lausanne, 21 in Experiment 1 (13 females, 18–28 years), and 17 in Experiment 2 (8 females, 19–31 years) took part in the study for a monetary reward (25 CHF/hour). In both experiments, we chose a sample size of convenience with the only criterion of aiming at more than 15 subjects, based on prior work reporting medium-to-large effect sizes (e.g., Cohen's *d’* > 0.5) in the strength of serial dependence with N ∼ 15 [28,29]. All participants had normal or corrected-to-normal vision and were naïve as to the purpose of the experiments. Visual acuity was tested with the Freiburg Acuity test [110]. Written informed consent was collected from all participants beforehand.

### 5.4 Stimuli and procedures

Fig. 1 illustrates the main aspects of the paradigm. Trials were organized into pairs, with alternating trials containing only the adjustment task (*no-load*) and trials including also the memory task (*load*). In Experiment 1, each trial of the *load* condition started with a fixation spot. After 500 ms, the fixation spot remained on screen while two abstract shapes were shown for 500 ms, at 6° on the left and right side of fixation. The two shapes were grey polygons generated via Delaunay's triangulation of a set of randomly determined points in space, with the constraint to always have ten sides. The choice of abstract shapes instead, for instance, of other-oriented stimuli, was made to avoid any systematic additive bias in orientation responses due to the potential similarity between adjustment and memory stimuli.

After a blank interval of 500 ms, a Gabor stimulus (peak contrast of 10 % Michelson, spatial frequency of 0.33 cycles-per-degree [cpd], and a Gaussian envelope of 0.75°) was presented at the center of the screen for 500 ms and followed by a noise mask (90 % contrast, 1000 ms duration). The Gabor shown on each trial could have any possible orientation within the 1:180° circular space, with the only constraint that the maximum absolute orientation difference between two consecutive Gabor stimuli was 40° (see Ref. [18,23,111] for similar approaches). The choice of restricting the space of orientation changes was motivated by the fact that attractive serial dependence in orientation usually peaks within the 10–40° range of orientation differences. Beyond this range, the effect tends to diminish or even transition to repulsion. Therefore, restricting orientation differences enabled us to focus on a range where attractive serial dependence is most pronounced, thereby increasing the number of trials within this relevant range [12].

After 250 ms, participants had to rotate a response bar to match the perceived orientation of the Gabor (adjustment task). The adjustment response was made by rotating the mouse in the upward and downward directions and confirming by pressing the left mouse button. Following the adjustment response, with a random interval between 500 and 1000 ms, participants were presented with a single central test shape. Their task was to report whether the test shape was one of the two shown on the initial memory display or not (old vs. new memory task) by pressing the ‘Y’ key for ‘old’ responses and the ‘X’ key for new responses.

The test shape was randomly chosen as one of the two shown on the memory display of the same trial or a different one from the set of available shapes. In *no-load* trials, no shape was presented either before or after the Gabor, but we included 1000 ms of a blank interval between the initial fixation and the Gabor and a random interval between 1 and 2 s after the adjustment response, to maintain approximately the duration and temporal relation between events as in the *load* condition. Trials of both conditions were separated by an inter-trial interval randomly determined within 1–2 s.

Experiment 2 was identical to Experiment 1 except that three, instead of two shapes were presented, placed at equidistant locations from fixation (see Fig. 1). As in Experiment 1, the three shapes could be identical (*low load*) or all different (*high load*). Both experiments consisted of 400 trials (10 blocks) for a total duration of approximately 1 h.

Before each experiment, participants were provided written and verbal instructions and performed a sequence of practice trials under the supervision of the experimenter. Practice trials were not analyzed further. Participants were instructed to maintain their gaze at the center of the screen for the entire duration (breaks excluded). All stimuli were presented on a grey background (62.66 cd/m^2^).

For the sake of clarity, it is worth noting that, while we have used terminology more traditional to the field of serial dependence in perceptual tasks, in the memory domain, the adjustment task used here is also known as the ‘recall,’ ‘delayed-recall,’ or ‘delayed reproduction’ task, whereas the shapes memory task is typically referred to as a ‘delayed recognition task’ with match/non-match responses. Similarly, the no-load condition refers to the absence of VWM load added to the load already required by the adjustment task (load of one stimulus only). As the goal of the study was to evaluate the effect of adding a VWM load task to the standard paradigm of serial dependence investigated in the perceptual domain, we maintain consistency with the terminology used in the serial dependence literature.

### 5.5 Analysis

At the individual trial level, outliers were removed using a two-step process. Initially, adjustment errors were calculated as the acute angle between the reported and true orientations, expressed in degrees. This computation was performed using the formula:

$$error={\left(\left(r-\theta \right)+90\right)}_{\mathrm{mod}\;180{}^{\circ}}-90$$

where r represents the reported orientation, θ is the actual stimulus orientation, and mod180° ensures that error values fall within the range of −90 to 90°.

Errors exceeding 45° in absolute value were identified as lapses and excluded from analysis. Subsequently, the remaining errors were demeaned, and any systematic biases in reporting orientations were removed by fitting a sum of six sinusoids using the MATLAB function *fit.m* with the model specification 'sin6' [23,28]. For outlier detection, errors greater than 1.5 interquartile ranges above the upper quartile or below the lower quartile were considered outliers and removed. This was done separately for each condition (*no-load*, *low load*, and *high load*) using the MATLAB function *isoutlier.m* with the method set to ‘quartiles’. Additionally, adjustment trials with durations exceeding 10 s were excluded from further analysis.

Overall, less than 15 % of trials were excluded (13 % in Experiment 1 and 10 % in Experiment 2), with participants completing the adjustment task within an average of 1.58 s in Experiment 1 and 1.38 s in Experiment 2.

As a performance measure in the orientation adjustment task, the error scatter, defined as the standard deviation of adjustment errors, was computed using the MATLAB function *std.m*. To ensure that the estimate of error scatter was not contaminated by the bias introduced by serial dependence, errors were also residualized from any effect of the orientation shown in the preceding trial. This additional residualization was done only for this specific analysis and was performed for each participant and load condition in the current trial, by residualizing errors from a moving average fit of the effect of previous stimuli (Δ).

In the analysis of recall performance, we compared the accuracy in different memory load conditions via a two-tailed paired *t*-test, followed by an estimate of the effect size (Cohen's *d’*) and Bayes factor analysis (BF_10_, evidence in favor of the alternative hypothesis).

In the analysis of serial dependence, we fitted a 1st derivative of a Gaussian function [10] to the adjustment errors as a function of the variable Δ, obtained as previous minus current orientation:

$$\Delta ={\left(\left(\theta_{n-1}-\theta_{n}\right)+90\right)}_{\mathrm{mod}\;180{}^{\circ}}-90$$

where $\theta_{n-1}$ and $\theta_{n}$ are the orientations of the stimulus shown on the preceding (n−1) and current trial (n), respectively. The δoG has the following form:

$$error=\Delta \alpha wce^{-{\left(w\Delta \right)}^{2}}$$

where $c=\frac{\sqrt{2}}{e^{-0.5}}$ is a constant and w is the inverse of the curve width. The half-amplitude parameter α quantifies the deviation of the errors, in degrees, from the actual orientation as a function of the Δ variable: positive values of α indicate a systematic deviation of errors towards the orientation of the preceding stimulus, and negative values indicate a deviation away —i.e., repulsion. The width of the curve indicates how narrow or wide is the effect of prior stimuli —i.e., the range of stimulus differences for which serial dependence occurs.

The parameters of the δoG function were estimated on the aggregate data of all participants, by solving a constrained non-linear minimization problem with the sum of squared residuals as the cost function (using *fmincon.m* in MATLAB). The initial parameters were set to α=2,w=0.05, and were refined through a search grid. The search constraints (upper, lower bounds) were α=[−20,+20],w=[0.01,0.1].

Statistical significance of the half-amplitude and width parameter was assessed via bootstrap resampling and surrogate null statistics, by randomly shuffling the sign of adjustment errors and comparing the observed parameters with the distribution of surrogate ones (N = 10000). Serial dependence between conditions was compared by randomly shuffling the group/condition labels 10000 times and comparing the distribution of the resulting differences against the observed one. In the figures, the average curves are depicted using folded errors, calculated by multiplying trial-wise errors by the sign of the trial-wise Δ. This approach is in line with recommendations from previous studies [112]. Given the symmetric nature of serial dependence patterns, folded errors for negative Δ are therefore represented as a mirrored and sign-flipped version of those in the positive range. It is important to note that this procedure is solely for graphical purposes, and all analyses, including model fit, were performed on the original error variable.

Where used, standard frequentist analyses (e.g., t-tests) were followed by estimates of effect size (Cohen's *d'*) and Bayes factor analysis (BF_10_, evidence in favor of the alternative hypothesis, testing one-tailed differences based on the direction of the effect). The Bayes factor analysis was based on the Scaled JZS Bayes factor using a Jeffrey-Zellner-Siow Prior with a default scale factor of 0.707 [113]. Cohen's *d'* measures the magnitude of the difference, categorized as small (around 0.2), medium (around 0.5), or large (0.8 and above). The Bayes factor (BF_10_) quantifies the evidence for H_1_ relative to H_0_, with BF_10_ < 1 favoring H_0_, 1 < BF_10_ < 3 indicating anecdotal evidence for H_1_, 3 < BF_10_ < 10 suggesting moderate evidence, 10 < BF_10_ < 30 showing strong evidence, 30 < BF_10_ < 100 indicating very strong evidence, and BF_10_ > 100 reflecting extreme evidence for H_1_. Conversely, BF_01_ is the inverse of BF_10_ and quantifies evidence for H_0_ relative to H_1_, with BF_01_ > 1 favoring H_0_. Discrepancies between p-values and Bayes factors can occur: a significant p-value with a low BF_10_ suggests a significant difference with weak evidence.

## Data availability

The datasets of this article are available in the Zenodo repository https://doi.org/10.5281/zenodo.12515047.

## CRediT authorship contribution statement

**Yuri A. Markov:** Writing – original draft, Visualization, Software, Methodology, Investigation, Formal analysis, Data curation, Conceptualization. **Natalia A. Tiurina:** Writing – original draft, Visualization, Supervision, Methodology, Investigation, Funding acquisition, Formal analysis, Data curation, Conceptualization. **David Pascucci:** Writing – original draft, Visualization, Supervision, Software, Project administration, Methodology, Funding acquisition, Formal analysis, Conceptualization.

## Declaration of competing interest

The authors declare that they have no known competing financial interests or personal relationships that could have appeared to influence the work reported in this paper.
