# Supplementary material for: Serial dependence: A matter of memory load
Source: Heliyon. 2024 Jul 2;10(13):e33977. doi: 10.1016/j.heliyon.2024.e33977 (PMC11283082; doi:10.1016/j.heliyon.2024.e33977)
Supplement: Multimedia component 1 [file mmc1.docx]

# Supplementary Material

## Control analysis with error binning

Our primary analysis of serial dependence involved fitting a function to the aggregate data of all participants, which is a common practice in the field (Fischer & Whitney, 2014; Fritsche et al., 2017; Pascucci et al., 2023). However, this approach has the potential drawback of overlooking inter-individual variability (Pascucci et al., 2019). To further validate our findings, we conducted a control analysis using arbitrary binning that took into account inter-individual variability. This method entailed subtracting the average error for positive Δ values from the average error for corresponding negative Δ values (Ceylan et al., 2021; Ceylan & Pascucci, 2023; Samaha et al., 2019), thereby obtaining an estimate of bias for each participant and condition, where positive values indicate attraction and negative values indicate repulsion from the previous orientation. We subsequently employed repeated-measures ANOVA to analyze these estimated biases.

### Results of Experiment 1

In Experiment 1, a two-way repeated-measures ANOVA was conducted with two factors: Trial (whether the VWM load manipulation was applied on the previous or current trial) and Load Level (*low* vs. *high*). This analysis revealed a significant main effect of Trial (F(1, 20) = 5.139, *p* = .034, $\eta_{p}^{2}$ = 0.204) indicating that the presence of a VWM load task on the current or previous trial influenced serial dependence. However, there was no main effect of Load Level (F(1, 20) = 0.002, *p* = .959, $\eta_{p}^{2}$ < 0.001), nor was there an interaction effect (F(1, 20) = 2.568, *p* = .124, $\eta_{p}^{2}$ = 0.113). These results align with the findings from the fitting analysis, suggesting that the level of VWM load in Experiment 1 did not reliably affect serial dependence.

### Results of Experiment 2

The same control analysis was performed on the results of Experiment 2, where a two-way repeated-measures ANOVA revealed a significant interaction between Trial and Load Level (F(1, 20) = 8.286, *p* = .010, $\eta_{p}^{2}$ = 0.341), thus supporting the conclusion that, with increasing load demand, the load level differentially modulated serial dependence, based on whether the load was imposed on the current or previous trial (see Figure 3CD in the main text).

## Controlling for adjustment performance in Experiment 2

In Experiment 2, increasing the load demand in the *high load* condition led to clear modulations of serial dependence. However, this was accompanied by inconclusive findings regarding the impact of load level on adjustment performance.

Anecdotal evidence of the effect of load level on adjustment performance may have arisen due to large interindividual variability, with some participants allocating attentional resources differently across load levels. For example, certain participants might have experienced greater 'distraction' when faced with a high load demand on the current trial, leading to reduced attention to the Gabor stimulus. Consequently, following a high-load trial where less attention was directed towards the Gabor, serial dependence may have decreased (Ceylan & Pascucci, 2023a, 2023b; J. Fischer & Whitney, 2014). Conversely, during trials with a high load demand, reduced attention to the current Gabor may have heightened the bias towards the past.

To control for these potential interindividual differences, we conducted an additional analysis where participants were split based on the effect of the load level on adjustment performance (median split of the difference in error scatter, *high* minus *low* *load*). We then evaluated the effects of manipulating the load level on the current trial and its impact on serial dependence across the two groups. If participants exhibiting increased error scatter under *high load* were indeed those driving the observed effect on serial dependence —e.g., because of reduced focus on the central Gabor, we would expect distinct patterns between the two groups. However, even participants who demonstrated highly comparable error scatter in both low and high load conditions displayed the same modulatory effect on serial dependence (group in the lower split, difference in the half-amplitude between the *low* and *high* *load* conditions = -1.03°, *p_perm_*  = .062; differences in width = -0.00, *p_perm_*  = .424; group in the higher split, difference in the half-amplitude between conditions = -1.14°, *p_perm_*  = .032; difference in width = -0.01, *p_perm_*  = .340; see Supplementary Figure 1), thus, ruling out the possibility that the results were merely driven by a subset of participants with specific attentional strategies.

Furthermore, this control analysis also highlighted different tendencies, with participants less affected by the load demand on adjustment performance exhibiting narrower effects of serial dependence, and those more affected by the load demand exhibiting a wider tuning of the effect of previous stimuli (difference in width between groups = 0.03 *p_perm_*  = .010), along with lower accuracy in the *high load* memory task (difference in VWM accuracy in the *high load* condition between groups: t(14) = 2.22, *p* = 0.043). Although not the primary focus of this study, these interindividual differences highlight distinct profiles in visual VWM and the featural tuning of serial dependence, which could be of interest for future research.

# Supplementary Figure 1


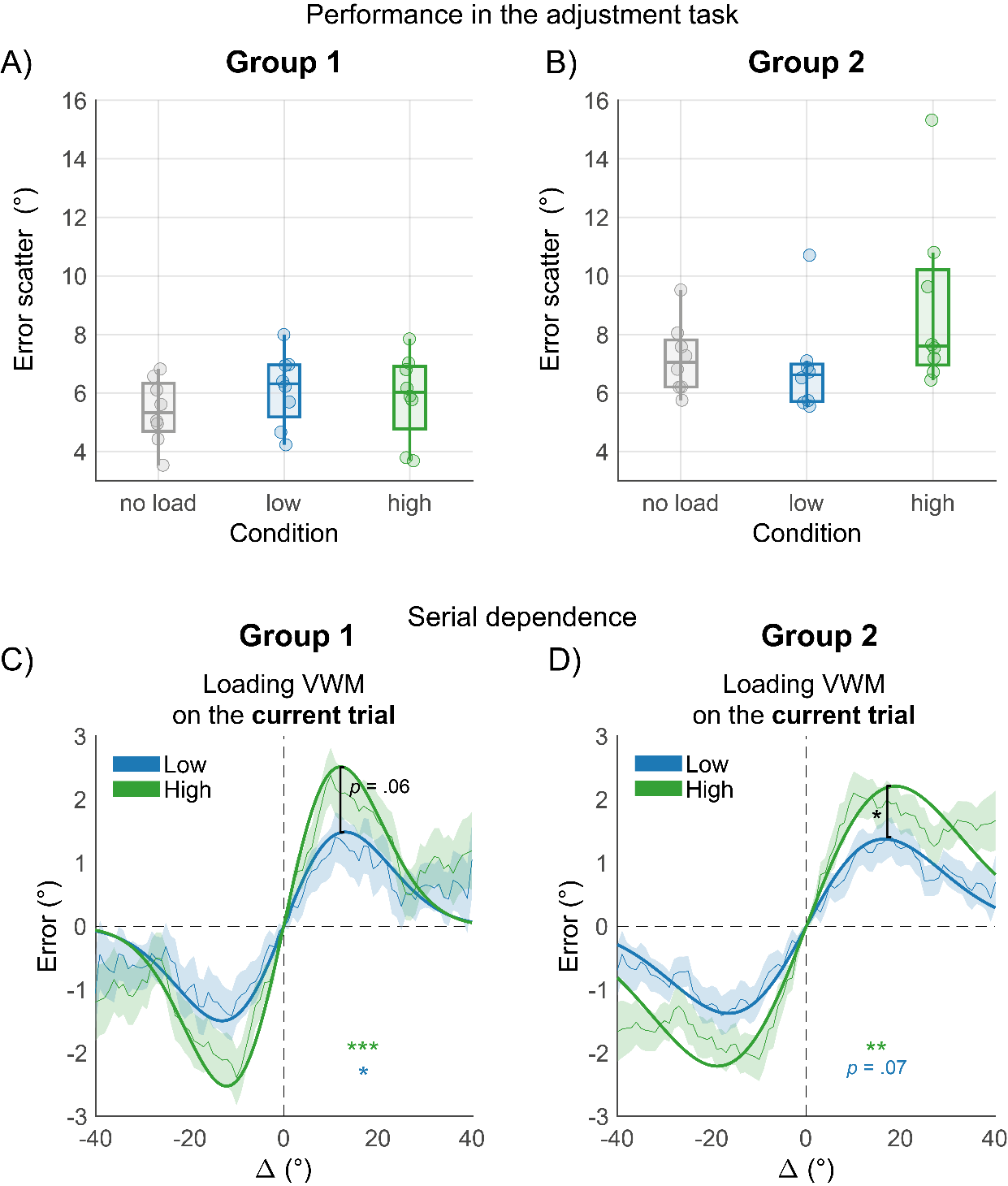


Results of the median splitting of participants in Experiment 2. A-B) The two groups were split depending on the difference in adjustment performance between the *low* and *high* *load* conditions. Group 1 represents the lower split, comprising participants with consistent performance levels across both conditions, while Group 2 represents the higher split, consisting of participants displaying larger performance differences and deterioration in the *high load* condition. C-D) Serial dependence was compared as a function of the load level on the current trial between the two groups. Both groups exhibited a similar increase in serial dependence under *high load*, despite Group 1 displaying entirely comparable adjustment performance across load levels. However, the two groups differed in the overall width of serial dependence, with participants more impacted by the load manipulation in their adjustment performance also demonstrating broader serial dependence (e.g., less tuning for similar orientations). The colors and elements of each panel are consistent with those depicted in the figures presented in the main text.
